# Statement of Retraction: Dihydroartemisinin represses oral squamous cell carcinoma progression through downregulating mitochondrial calcium uniporter

**DOI:** 10.1080/21655979.2025.2491924

**Published:** 2025-06-20

We, the Editors and Publisher of the journal *Bioengineered*, have retracted the following article:

Zheng, S., Wu, R., Deng, Y., & Zhang, Q. (2021). Dihydroartemisinin represses oral squamous cell carcinoma progression through downregulating mitochondrial calcium uniporter. *Bioengineered, 13*(1), 227–241. https://doi.org/10.1080/21655979.2021.2012951

Since publication, significant concerns have been raised about the integrity of the data and reported results in the article. When approached for an explanation, the authors have fully cooperated with the investigation and have provided additional data and information.

However, the concerns remain unaddressed. As verifying the validity of published work is core to the integrity of the scholarly record, we are therefore retracting the article. The corresponding author listed in this publication has been informed. The corresponding author disagrees with the retraction.

We have been informed in our decision-making by our editorial policies and the COPE guidelines.

The retracted article will remain online to maintain the scholarly record, but it will be digitally watermarked on each page as ‘Retracted’.

